# Annotations

**Published:** 1910-09-10

**Authors:** 


### Annotations.

### Opium Dens in France.

There has recently been tried at Brest a case in which, as the result of opium smoking, two young women had died, presumably from narcotic poisoning. The prisoners were four ladies of easy virtue, a photographer, and a pharmacist's assistant, who were prosecuted for the illegal possession of, and traffic in, opium. After hearing the defence the judge condemned each of the women to a month and each of the men to forty days' imprisonment, and in addition each prisoner was fined 100 francs, stay of execution being refused. The public prosecutor in his address bore eloquent witness to the pernicious effects on the *morale* of the army and navy caused by the opium habit, and the commissioner appointed to inquire into the case in addition gave out that 40 per cent. of the officers in the Brest garrison were habitual opium smokers. There may, of course, be some exaggeration in these statements, but the *Journal de Médecine*, to which we owe the account of the case, thinks it extraordinary that the authorities should have thought it worth while to prosecute these wretched women, who after all were but "supers" in the tragedy, while the real actors remain unpunished. The opium dens are well known to all the officers, and it would need but a slight display of official energy to close them all. Sufficiently severe penalties could then be devised to put a stop to the trade of the importers of the drug. It is probable that the Chinese will be rid of their opium dens long before Europe will be of its dens.

### Mantegazza and James.

The deaths of Professors Paolo Mantegazza and William James will be regretted by many to whom these scientists have been more than mere names during the past decade. The Italian was essentially an interpreter, whose mission in life appeared to be chiefly to popularise certain departments of medicine which have always been overlaid by a veneer of polished professionalism. A widely read man, in private life the most charming of companions and the staunchest of friends, Mantegazza made a friend of everyone with whom he came into close touch, and though his convictions were proclaimed with a loudness of tone and a disregard of convention that laid him open to criticism and attack, he was undoubtedly one of the most popular professors of the day. His work in anthropology and social science is a lasting memorial to his genius, and deserves to be much more widely known in this country than it is at present. Far more generally appreciated, by those at least who did not differ so fundamentally from him as to be incapable of honest admiration, was Professor William James, whose connection with Harvard has given that University a reputation, so far as experimental psychology is concerned, which transcends that of any American seat of learning. Popularly, but erroneously, regarded as the founder of Pragmatism, James won an enduring place in the front rank of men of science by his sterling good work—much of it essentially pioneering—in the most fascinating, though still the most nebulous, of sciences. This is not the place to speculate as to his indebtedness to contemporaries and predecessors, but it may safely be affirmed that the wealth of original thought that is to be found in his writings will ensure him a lasting place among the great philosophers of the latter end of the nineteenth century, and that medicine in the near future will increasingly recognise the value of his contributions to the study of the human mind.

### A Note on Parisian Hospitals.

Mr. A. R. Thompson, the surgeon in charge of the newly created genito-urinary department at Guy's Hospital, who has been studying Continental methods in connection with his department, contributes an interesting series of notes to the current issue of the Guy's Hospital *Gazette* on his observations in the Paris hospitals. Like most English surgeons who visit the French institutions, Mr. Thompson was struck with the discrepancy between the "official content" of the wards, as indicated by the legend affixed to the wall and the actual number of occupants, but he qualifies the inevitable adverse criticism by remarking that the air appeared to be perfectly fresh, and that the wards possess an unusually large amount of window space. The nursing service impressed him very unfavourably. "The nurses," he remarks, "were not anywhere near the same standard of the nurses in London. Coarse-looking and dirty for the most part, none apparently with any great or defined knowledge, some of them even struck me as being more suitable for the work of charing than that of nursing." But above all his attention was directed to the slovenly manner in which anæsthetics were administered, though he pays a well-deserved tribute to the excellence of the arrangements at the Beaujon, where Professor Tuffier operates. "The administration of chloroform, which is commonly used," he states, "seems to be governed by the following simple rule: 'Continue administering the chloroform, in large quantities and at regular intervals, until you are quite certain that the patient is not breathing: if in any doubt, continue the administration!'" Dealing with the patients at the surgical clinics, he remarks that they appear to be much more resolute in enduring pain, and much more interested in their own complaints, than the average English patient. "Their resolution in enduring pain is wonderful. This fact is a tacit admission of the badness of the administration of anæsthetics." At the Beaujon patients who pay £20 a year rent are expected and bound to pay for treatment as in-patients, the charge varying from four to five francs per week. Post graduate students who are desirous of trying one or other of the Parisian clinics will find many helpful and valuable hints in Mr. Thompson's paper.

